# Next-Generation Sequencing for Bloodstream Infections: Shaping the Future of Rapid Diagnostics and Precision Medicine

**DOI:** 10.3390/diagnostics15232944

**Published:** 2025-11-21

**Authors:** Ayman Elbehiry, Eman Marzouk, Husam M. Edrees, Moustafa H. Abdelsalam, Feras Aljizani, Saad Alqarni, Eyad Khateeb, Feras Alzaben, Mai Ibrahem, Ayman M. Mousa, Nasser Huraysh, Akram Abu-Okail

**Affiliations:** 1 Department of Public Health, College of Applied Medical Sciences, Qassim University, P.O. Box 6666, Buraydah 51452, Saudi Arabia; e.marzouk@qu.edu.sa; 2 Department of Physiology, Faculty of Medicine, University of Tabuk, Tabuk 47191, Saudi Arabia; hedrees@ut.edu.sa (H.M.E.); mnosseir@ut.edu.sa (M.H.A.); 3 Department of Medical Laboratory, King Fahad Armed Forces Hospital, Jeddah 23311, Saudi Arabia; 4 Department of Family Medicine, King Fahad Armed Forces Hospital, Jeddah 23311, Saudi Arabia; 5 Department of Food Service, King Fahad Armed Forces Hospital, Jeddah 23311, Saudi Arabia; 6 Department of Public Health, College of Applied Medical Science, King Khalid University, Abha 61421, Saudi Arabia; 7 Department of Basic Health Sciences, College of Applied Medical Sciences, Qassim University, Buraydah 51452, Saudi Arabia; 8 Department of Pathology and Laboratory Diagnosis, College of Veterinary Medicine, Qassim University, Buraydah 51452, Saudi Arabia

**Keywords:** bloodstream infections, sepsis, metagenomic sequencing, microbial cell-free DNA, nanopore, antimicrobial resistance, genomic epidemiology, public health

## Abstract

Bloodstream infections and sepsis necessitate rapid, sensitive, and clinically relevant diagnostics to minimize treatment delays and improve clinical outcomes. Next-generation sequencing enables culture-independent pathogen detection, antimicrobial resistance profiling, and genome-informed epidemiology. This narrative review integrates clinical evidence with practical workflows across three complementary approaches. We describe the use of plasma microbial cell-free DNA for broad organism detection and burden monitoring, as well as metagenomic next-generation sequencing of blood or plasma for unbiased pathogen discovery, including culture-negative and polymicrobial infections. Same-day Oxford Nanopore Technologies sequencing of positive blood culture broth is also discussed as a way to accelerate species identification, targeted resistance reporting, and infection-prevention decisions. We outline the sample-to-result steps, typical turnaround time (TAT), and stewardship-aligned decision points. Analytical reliability depends on effective reduction in human DNA background, stringent control of background and reagent-derived nucleic acids in low-biomass samples, and documented and validated bioinformatics workflows that are supported by curated taxonomic and resistance databases. Quantitative reports should adhere to validated thresholds and should be interpreted in the context of internal controls and clinical pretest probability. Ongoing challenges include variable correlation between genotype and phenotype for specific pathogen and antibiotic pairs, interpretation of low-level signals, and inconsistent regulatory and reimbursement environments. Advances in portable sequencing, faster laboratory and analytical workflows, and scaled liquid biopsy strategies may further reduce the TAT and expand access. Integrating these tools within One Health frameworks and global genomic surveillance programs could support early resistance detection and coordinated public health action, which could help to advance sepsis care toward more precise treatment and real-time infection control insights.

## 1. Introduction

Bloodstream infections (BSIs) and sepsis are among the most urgent problems associated with infectious diseases and cause major global morbidity, mortality, and economic burdens [1,2,3]. The Global Burden of Disease (GBD) analysis estimated that 48.9 million incident sepsis cases and 11.0 million deaths occurred in 2017, which corresponds to nearly one in five deaths worldwide, with the highest burden being reported in Sub-Saharan Africa and South and Southeast Asia [4]. The Sepsis-3 definition describes sepsis as life-threatening organ dysfunction resulting from a dysregulated host response to infection, which is identified clinically by an acute increase of 2 points or more in the Sequential Organ Failure Assessment (SOFA) score [5,6,7]. According to a 2024 report from the World Health Organization (WHO), early recognition and timely treatment remain essential to prevent progression to septic shock and multiorgan failure, particularly in low- and middle-income countries [8].

Blood culture (BC) continues to be the diagnostic standard, but it is slow (often requiring hours to days), and its sensitivity is adversely affected when performed after antibiotic administration and for patients infected with fastidious or intracellular organisms [9,10]. Antibiotic pretreatment significantly decreases BC yield, which reinforces the need for faster culture-independent approaches [11]. Timely delivery of appropriate antimicrobial therapy is strongly associated with survival, which has led the Surviving Sepsis Campaign to emphasize rapid identification of the infectious source and prompt selection of effective treatment [12].

Bacterial antimicrobial resistance (AMR) is an urgent and escalating global health threat. According to the GBD 1990–2021 AMR Collaborators (GRAM), an estimated 4.71 million deaths in 2021 were associated with bacterial AMR, including 1.14 million deaths directly caused by resistant infections [13]. The analysis also revealed that by 2050, the highest all-age AMR mortality rates will be concentrated in South Asia, Latin America, and the Caribbean [13]. For comparison, the GBD 2019 baseline analysis estimated that there were 4.95 million AMR-associated deaths and 1.27 million AMR-attributable deaths in 2019, which underscores that the burden remains substantial and persistent [14].

Next-generation sequencing (NGS), including metagenomic next-generation sequencing (mNGS), has emerged as a culture-independent tool for identifying suspected BSIs [15,16,17]. mNGS can identify bacteria, DNA viruses, fungi, and parasites in a single analysis, although foundational work shows that strict preanalytical measures and validated bioinformatics methods are essential for reliable interpretation [18,19]. Across BSI and sepsis cohorts, mNGS frequently outperforms culture in pathogen detection, particularly after antibiotic exposure or when fastidious or mixed infections are suspected, and it often provides clarifying information that influences therapy [20].

A major recent advance is the use of plasma microbial cell-free DNA (mcfDNA), which is fragmented circulating microbial DNA that is released during infection. Clinical validation studies have demonstrated broad detection covering approximately 1000 to 1250 clinically relevant pathogens, with quantitative reporting of molecules per microliter (MPM) [21]. Higher quantitative mcfDNA levels are consistently correlated with confirmed infection and treatment response, which supports the use of quantitative thresholds in interpretation [22].

In addition to mcfDNA, shotgun mNGS for blood or plasma samples provides unbiased pathogen detection and has shown greater diagnostic yield than culture in several cohorts. Targeted sequencing methods such as 16S ribosomal RNA gene analysis, internal transcribed spacer (ITS) sequencing for fungi, and focused AMR panels can shorten the turnaround time (TAT) and increase sensitivity when microbial reads are limited by the host DNA background [23]. Real-time long-read platforms, particularly Oxford Nanopore Technologies (ONT) sequencing, have enabled same-day pathogen identification and early inference of AMR determinants from either positive BC broth or (in some workflows) directly from blood or plasma. Multiple studies have evaluated the accuracy, reproducibility, and standardization of these methods and support their growing clinical use [24]. National networks are also evaluating sequencing for rapid pathogen screening and public health surveillance [25,26].

Bacterial BSIs are responsible for most sepsis cases and drive a considerable share of the AMR burden, which has led to pathogen genomics becoming central to patient management and infection control [27,28]. Whole-genome sequencing (WGS) and targeted assays can differentiate species and lineages and can detect clinically important AMR markers. These markers include the carbapenemases *bla*KPC, *bla*NDM, and *bla*OXA-48-like genes; extended-spectrum β-lactamases (ESBLs) such as *bla*CTX-M-15; and glycopeptide resistance genes *vanA* and *vanB*. These capabilities support early escalation or de-escalation of therapy and facilitate high-resolution outbreak investigations [29]. Recent BSI studies and national surveillance programs have demonstrated that WGS can delineate the plasmid context of carbapenemases, define ESBL gene distribution patterns, and confirm van gene carriage in vancomycin-resistant (VRE) and vancomycin-variable enterococci (VVE) during routine monitoring [30].

ONT now enables practical core genome multilocus sequence typing (cgMLST) and single-nucleotide polymorphism (SNP) analysis, although variations in chemistry, base calling, allele calling, and clustering thresholds can affect relatedness interpretation. This highlights the need for validated and standardized pipelines with transparent cutoffs [31]. For viral and fungal BSIs, sequencing contributes to the characterization of drug resistance, including human immunodeficiency virus (HIV), hepatitis B virus (HBV), and hepatitis C virus (HCV) genotyping, as well as species-level identification of candidemia and cryptococcemia. Such methods are increasingly being applied in azole resistance assessment [18].

Several practical challenges remain. Blood samples have low biomass and are dominated by host DNA, but host depletion or pathogen-enrichment protocols can improve microbial read recovery and analytical sensitivity [32]. Reagent and environmental contamination, index misassignment, and variable reference databases may compromise interpretation, and best practices include the use of extraction blanks, batch modeling, and contamination-aware computation [33]. Clinical interpretation should incorporate quantitative metrics such as MPM trends, host factors, imaging findings, and conventional microbiology to distinguish infection from colonization or transient translocation, as well as to avoid misinterpretation of residual or nonviable microbial DNA [34]. Adoption is influenced by economic and regulatory considerations, including reimbursement and laboratory accreditation, and current Surviving Sepsis guidelines endorse rapid but stewardship-aligned use of antimicrobials, which sequencing-based diagnostics can directly support [35].

In this review, we summarize the current role of NGS in BSIs with a primary focus on bacterial disease because of its central contribution to sepsis and AMR. Viral and fungal infections are also addressed. We outline the evolution of sequencing platforms and clinical workflows and clinical evidence supporting mNGS and plasma mcfDNA in suspected BSI, including culture-negative and polymicrobial disease. We also discuss key preanalytical, analytical, and interpretive challenges, such as host depletion, contamination control, quantitative thresholds, and AMR detection, as well as future directions, including point-of-care real-time sequencing, liquid biopsy approaches, and One Health genomic surveillance. Section 3.5 consolidates detailed information on TATs and clinical indications.

## 2. Search Strategy and Selection Criteria

We performed a structured literature search in PubMed/MEDLINE, Embase, Web of Science, Scopus, and the preprint servers medRxiv and bioRxiv to identify studies published from January 1998 to September 2025. The search strategy used the following terms: (“bloodstream infection” OR sepsis) AND (metagenomic OR “next-generation sequencing” OR “microbial cell-free DNA” OR nanopore OR “Oxford Nanopore” OR “long-read” OR “whole-genome sequencing”) AND (diagnostic OR “antimicrobial resistance” OR stewardship). These terms are reported in full to reflect the exact database queries. Filters were applied to restrict the results to human studies and English-language publications.

Eligible studies included original research (prospective or retrospective cohorts and clinical trials), systematic reviews and meta-analyses, methodological and analytical validation studies, and authoritative guidelines or consensus statements. To qualify for inclusion, studies had to report at least one of the following: diagnostic performance, turnaround time (TAT), clinical utility, or implementation considerations related to NGS, mNGS, plasma mcfDNA, ONT, WGS, AMR, or antimicrobial stewardship (AMS). We excluded single case reports without methodological relevance, animal-only studies, purely in silico or bioinformatics evaluations lacking clinical specimens, and non-peer-reviewed opinion pieces. The reference lists of all included studies were manually reviewed to identify additional relevant publications.

During early drafting, we used an AI-assisted language tool only to help refine the wording of a few sentences. All scientific content was conceived, analyzed, and written by the authors, and the manuscript was subsequently revised and edited in full by the author team without further use of AI tools.

## 3. Technologies and Workflow for Sequencing in BSIs

This section integrates three complementary sequencing approaches used in suspected BSIs: plasma mcfDNA, direct metagenomic sequencing of blood or plasma, and rapid nanopore sequencing from positive BCs. For each approach, we summarize preanalytical considerations, the breadth of organism and AMR detection, the TAT, and how results guide escalation, de-escalation, or targeted source evaluation.

### 3.1. From Short-Read NGS to Real-Time Long Reads

Clinical sequencing has progressed from Sanger single-locus assays to NGS and, more recently, long-read platforms. Short-read instruments (primarily Illumina) remain the foundation of most mNGS workflows because of their high throughput, stable per-base accuracy, and ability to process large sample batches, enabling hypothesis-free detection directly from clinical specimens [18,36].

Long-read instruments provide faster results and richer genomic context. ONT enables same-day species identification and AMR gene inference directly from positive BC broth, supported by prospective and methodological studies showing accurate identification and on-target AMR detection. Adaptive sampling (software-directed selective sequencing to enrich target DNA) can further improve pathogen coverage [24,37,38]. Multiple studies have demonstrated sufficient accuracy and reproducibility for cgMLST and SNP-based relatedness analyses, supporting pragmatic hospital typing workflows [39]. At the platform level, PacBio HiFi chemistry delivers long reads with approximately 99.9% consensus accuracy, facilitating plasmid and repeat resolution and enabling nearly complete assemblies [40,41,42,43].

Short reads remain optimal for deep, unbiased metagenomics and quantitative reporting, whereas long reads compress turnaround and support plasmid reconstruction and near real-time typing. Many centers combine both short-read mNGS for broad, direct-from-sample detection and rapid ONT for same-day identification and AMR reporting after BC positivity [18,24,39]. Short- and long-read hybrid assemblies often yield the most reliable plasmid reconstructions and mobile resistance contexts, enhancing outbreak investigations and transmission mapping [29,44].

### 3.2. Sample to Answer Routes in Suspected BSI

#### 3.2.1. mcfDNA (Liquid Biopsy)

mcfDNA sequencing is minimally invasive and detects pathogens even when cultures are negative. A leading quantitative assay has demonstrated validated analytical and clinical performance across approximately 1250 clinically relevant pathogens, reported in MPM [21]. Real-world series (>15,000 patients) demonstrate broad organism recovery and clinically relevant impacts, especially in culture-negative or antibiotic-pretreated cases [22]. Because the results are quantitative, programs should predefine interpretation thresholds and serial trend criteria (e.g., percent change in MPM over time) and link them to specific clinical actions [22,45].

Diagnostic performance is especially strong in immunocompromised hosts. In suspected pneumonia, adding plasma mcfDNA testing to conventional diagnostics significantly increased yield and influenced antimicrobial choices and source investigations [46]. Similar findings have been described in solid organ transplant (SOT) recipients, where informed escalation or de-escalation has been tested and targeted diagnostic evaluation has been prompted in a substantial proportion of cases [47]. mcfDNA can persist longer than BC positivity in some bacteremic or partially treated endovascular infections. This extended detection window helps identify infection after early antibiotic exposure and may even suggest a risk of metastatic spread [48].

Interpretation requires clinical context. mcfDNA does not specify the anatomic source and does not provide phenotypic minimum inhibitory concentrations (MICs); therefore, integration with imaging, source evaluation, and local susceptibility patterns is essential [49]. Serial quantitative values and predefined thresholds help separate clinically meaningful signals from transient or residual DNA [50]. Health-system assessments indicate that these tests are most valuable when cultures are negative or unobtainable or when fastidious or occult pathogens are suspected. They are also useful when sequential quantitation helps distinguish active infection from mere translocation.

Cost analyses in selected high-risk groups suggest that early incorporation of mcfDNA testing may be economically reasonable when it shortens diagnostic pathways or reduces invasive testing [51]. Many hospitals embed structured stewardship pathways so that predefined test results trigger standardized optimization of antimicrobial regimens and targeted diagnostic steps within infectious diseases/intensive care unit (ID/ICU) services [52].

Two considerations merit emphasis. First, because blood is a low-biomass matrix, residual or nonviable microbial DNA can still generate signals. Interpretation should therefore incorporate the clinical context, and serial MPM trends with predefined quantitative thresholds can help limit overcalling [50]. Second, although many cohorts demonstrate clear clinical utility, some reports describe variable yields in specific settings, highlighting the need for careful test selection and multidisciplinary review [53]. Overall, the use of plasma microbial cell-free DNA expands the diagnostic toolkit for suspected BSI, especially in culture-negative, pretreated, or immunocompromised presentations, by combining broad organism coverage with quantitative reporting and detection kinetics that support timely, targeted care [22]. The detailed turnaround ranges and best-fit indications appear in Section 3.5.

#### 3.2.2. Direct Metagenomics from Blood or Plasma

Direct metagenomic sequencing of whole blood or plasma enables unbiased detection of bacterial, viral, fungal, and parasitic nucleic acids and can reveal polymicrobial infections or unexpected organisms. Clinical series and reviews show added diagnostic yield, and in selected contexts, the sensitivity can exceed that of BC; performance varies by matrix, sampling time, and host status [54]. Preanalytical handling has a major effect on yield.

Collect an adequate volume in EDTA tubes, separate plasma promptly, and avoid repeated freeze–thaw cycles, as these factors influence mcfDNA stability and recovery [55,56,57]. Because host DNA dominates these samples, sensitivity depends on validated host-depletion or pathogen-enrichment strategies. These approaches must be verified on the target matrix to ensure that microbial signals are preserved (see Section 5.1) [58].

Contamination control is particularly important for low-biomass blood and plasma samples. Extraction blanks, run-level negative controls, and contamination-aware background models help distinguish true pathogen signals from reagent or environmental DNA. Nonredundant unique dual indexing (UDI) with post-run filtering can further mitigate index cross-talk on patterned flow cell instruments (see Section 3.4 for detailed quality safeguards) [33,59,60].

Analytical reporting requires quantitative standards. Human-read subtraction, predefined detection thresholds, and interpretation relative to background models and internal controls help distinguish true infection signals from noise. Laboratories should specify quantitative units and cutoffs such as reads per million (RPM) or unique k-mers per organism and align these thresholds with background models and negative controls [61].

Diagnostic performance depends on the clinical context. For plasma mcfDNA, yield is often greater in pretreated or immunocompromised patients, with effect sizes differing by syndrome and supported by prospective and multicenter data where available [62]. For cerebrospinal fluid (CSF), metagenomic sequencing improves detection in culture-negative central nervous system infections when thresholds and controls are predefined. Applying contamination-aware models further strengthens interpretation [63,64].

For rapid nanopore sequencing from positive BCs, same-shift workflows can provide species-level identification and AMR insights within hours, with high concordance compared with routine methods. Any discordant results should undergo phenotypic confirmation [24]. Because blood, plasma, and CSF share low microbial biomass, interpretation should account for matrix-specific background and follow the contamination-control principles outlined in Section 3.4 and Section 5.1 [33,59,65].

Implementation should prioritize matrix-specific verification of host depletion and extraction, as well as the use of extraction blanks and negative controls in each batch. Quantitative thresholds should be explicitly linked to background models and clinical context, rather than creating separate workflows for each syndrome. Sequencing outputs from direct blood or plasma metagenomics should be interpreted alongside clinical data, including host response, imaging, and disease course. TAT and best-fit indications are summarized in Section 3.5 [18,58].

#### 3.2.3. Rapid Sequencing of Positive BCs

Once a BC becomes positive, sequencing the broth can markedly shorten the time to organism identification and genotypic AMR prediction. Prospective ICU cohorts show that ONT workflows can provide species-level identification within hours and support genotypic resistance inference by detecting key AMR genes. They can also characterize clinically relevant plasmids and resistance loci directly from culture material [24]. Early studies demonstrated real-time detection of pathogens, plasmids, and resistance determinants from positive bottles. Newer protocols incorporate adaptive sampling to enrich pathogen reads and further improve turnaround and genomic coverage [37].

Parallel multicenter evaluations have examined the accuracy and reproducibility of ONT-based bacterial typing using cgMLST and SNP analysis. These studies help define validation requirements, operational controls, and mitigation measures needed for clinical implementation [39]. To support same-day clinical decision-making, laboratories should prespecify reportable endpoints, including organism identification, priority resistance markers, and plasmid carriage. These results should then undergo phenotypic confirmation, with timely notification of infection-prevention teams.

These data support a pragmatic role for ONT in suspected BSI. Rapid sequencing from positive cultures can accelerate targeted treatment adjustment and guide infection-prevention interventions, whereas short-read or hybrid sequencing may be reserved for high-resolution epidemiology or near-complete assemblies [24]. The TATs and clinical indications for this approach are summarized in Section 3.5.

### 3.3. Targeted Sequencing: Focused Speed and Depth

While shotgun mNGS maximizes breadth, targeted sequencing concentrates reads on informative loci, increasing analytical sensitivity and shortening the TAT when the host background is high or the clinical question is narrow [18]. Common approaches include single-locus barcoding, such as 16S ribosomal RNA gene sequencing for bacteria and internal transcribed spacer (ITS) sequencing for fungi; multiplex amplicon panels that report species and resistance markers; and hybrid-capture enrichment to retrieve microbial sequences from complex blood matrices. These strategies provide faster, deeper results when unbiased metagenomics is unnecessary or not sensitive enough [18].

This longstanding clinical experience shows that 16S sequencing assists in the identification of difficult-to-culture or phenotypically atypical bacteria and adds value in polymicrobial or culture-negative disease. It is especially helpful for aerobic actinomycetes, anaerobes, and other fastidious organisms present at low abundance. Classic and contemporary reviews summarize key limitations, including copy-number variation, incomplete species-level resolution for specific genera, and dependence on well-curated reference databases. They also show that full-length 16S sequencing with long-read platforms improves taxonomic discrimination and supports routine workflows [66].

For fungi, the internal transcribed spacer region is the primary DNA barcode and offers a high probability of correct identification across broad taxonomic groups. Quality-controlled databases and careful primer selection mitigate amplification bias, which is critical in low-biomass blood specimens and mixed infections. In candidaemia and other invasive mycoses, ITS sequencing complements culture by accelerating species-level calls that shape antifungal selection and epidemiologic assessment [67]. For molds and cryptic yeasts, supplemental loci such as the D1/D2 domains of 28S rDNA, translation elongation factor 1 (TEF1), and beta-tubulin can resolve complexes that the internal transcribed spacer alone cannot [68].

Beyond single loci, targeted NGS (tNGS) panels focus on clinically actionable markers, including ESBLs and carbapenemases in Gram-negative organisms, glycopeptide resistance in enterococci, and azole or echinocandin resistance determinants in *Candida*, such as ERG11 and FKS1/FKS2. Bloodstream-oriented amplicon workflows can rapidly detect pathogens and key resistance loci. Capture-based enrichment increases target yield from blood, with reports of higher detection rates than BC alone in suspected BSIs [69]. When the host background is substantial, capture-based enrichment can markedly increase microbial signals. Validation should also account for off-target capture and allelic dropout.

The integration of resistance markers still requires clinical judgment. In yeasts, azole resistance often reflects target-site substitutions or efflux upregulation, and echinocandin resistance is associated with FKS hotspot mutations. Targeted assays can detect and trend these variants. As with bacteria, translation from genotype to phenotype depends on the organism and clinical context and is strengthened by curated knowledge bases and confirmatory testing when results conflict with the clinical course [70].

In practice, targeted assays are preferred when a defined set of organisms or mechanisms is suspected, when rapid deep coverage of specific loci is likely to change therapy, or when the host DNA burden limits unbiased mNGS sensitivity. Many laboratories adopt a hybrid strategy, using targeted sequencing for fast, actionable identification and AMR calls, and reserving shotgun mNGS or WGS for broad discovery, outbreak investigation, or comprehensive resistome and virulome profiling [71]. Timing and indication guidance are centralized in Section 3.5.

### 3.4. Bioinformatics, Reporting, and Quality Safeguards

Across platforms, rigorous bioinformatics validation and transparent reporting are essential. Key analytical decisions include human-read removal, appropriate classifier selection, use of curated reference databases, annotation of AMR determinants, and integration of quantitative sequencing results with the clinical picture (laboratory findings, imaging, pretest probability). Together, these factors directly influence analytical validity and clinical utility [18]. In addition, pipelines used for clinical reporting should be fixed and clearly document the software and database versions. Any update should trigger regression testing and partial revalidation, and reports should disclose the software/database versions and key run metrics to support interpretation and reproducibility [72].

Because blood is a low-biomass matrix, contamination-aware computation and statistics are central to accurate interpretation. The Run-level, Index-aware Decontamination and Evaluation (RIDE) framework emphasizes processing extraction blanks and run-level negative controls alongside clinical samples and tracking batch effects. It applies background-informed models to separate authentic pathogen signals from DNA introduced during handling or through index cross-talk in multiplexed runs [33].

Patterned flow cell instruments may show index misassignment. Mitigations include unique or nonredundant dual indexing combined with post-processing filters that remove unexpected index combinations. Laboratories should document their indexing strategy and misassignment thresholds in their validation records [65]. Where feasible, laboratories adopt UDI and apply post-run index-swap filters. UDI substantially reduces index hopping and enables automated removal of hopped reads during demultiplexing [73].

Host-DNA depletion or pathogen-enrichment approaches must be validated specifically for low-biomass blood or plasma matrices to ensure that sensitivity is preserved. Because mcfDNA is often scarce in BSIs, assay sensitivity may scale with input volume. Processing larger plasma volumes can increase pathogen cfDNA recovery and detection probability, although overall performance also depends on extraction efficiency, sequencing depth, and host-DNA background [49,74]. Even after depletion, microbial DNA may persist at <1:1000 relative to host DNA, underscoring the need for method verification [33,75,76].

Each analytical batch should include extraction blanks, run-level negatives, and periodic positives, with documentation of taxa detected in negatives to identify potential contamination pathways. Current consensus favors applying controls and mitigation measures end-to-end, from collection and extraction through library preparation, indexing, sequencing, and analysis [33,76]. Practically, this means predefining contamination-screen pass/fail gates (for example, the maximum allowable signal of common kit contaminants in negative controls) and recording corrective actions in the validation record [33,77].

The bioinformatics pipeline should incorporate human-read subtraction with appropriate privacy controls and mitigation measures, as well as contamination-informed background models derived from blanks and negative controls. It should also use fixed, documented taxonomic and AMR classifiers that are supported by curated database builds. Reporting should include run-level metrics (total reads, percent human, microbial reads per target), internal control performance, and software and database versions to ensure interpretable, reproducible results that support stewardship-aligned decisions [76,78,79,80].

Taxonomic and resistance calls should rely on well-curated databases and fixed, documented workflows. Benchmarking supports the use of k-mer classifiers (for example, Kraken2 with Bracken or Centrifuge). The choice should be guided by trade-offs in speed, memory, and specificity, with prespecified confidence thresholds and documented database-build dates [80,81]. Whichever tool is selected, software versions, reference builds, and decision thresholds must be defined before clinical implementation and updated only with revalidation [82].

AMR annotation tools, including the Comprehensive Antibiotic Resistance Database (CARD) with Resistance Gene Identifier (RGI), ResFinder with PointFinder, and AMRFinderPlus, differ in their curation strategies and variant coverage. When reporting genotypic resistance, laboratories should state identity and coverage cutoffs and any species-specific mutation rules, and note that genotype and phenotype may diverge. Any conflicts should be flagged for confirmatory testing [83,84,85].

Minimum validation elements for clinical deployment include matrix-specific limits of detection, within-run and between-run precision, and reproducibility across operators. Additional requirements include validation of human-read subtraction with appropriate privacy controls and mitigation measures, along with prespecified quantitative reporting parameters (for example, RPM or MPM) accompanied by clear interpretation guidance. Under Clinical Laboratory Improvement Amendments (CLIA) and College of American Pathologists (CAP) frameworks for clinical metagenomics, validation designs emphasize internal controls, orthogonal confirmation, and transparent documentation [61,72].

Reporting frameworks such as Strengthening the Reporting of Observational Studies in Epidemiology (STROBE)-metagenomics and the Enhancing the Quality and Transparency of Health Research (EQUATOR) Network guidance support comprehensive, transparent documentation of specimen handling, sequencing, and bioinformatics analysis. These frameworks apply to both validation studies and real-world clinical settings [86].

To assist in practical adoption, Table 1 summarizes widely used bioinformatics resources for bloodstream sequencing, including their evidence-supported capabilities, primary outputs, and documentation requirements. These examples are tool-agnostic and are intended to guide laboratory implementation without prescribing specific platforms. These elements define the core analytic safeguards, while Section 5.2 focuses on how variation in classifier, database, and threshold choices between laboratories affects comparability.

As laboratories adopt long-read sequencing for cgMLST and SNP typing, plasmid reconstruction, and outbreak investigation, they should define and validate platform chemistry, base-calling models, allele-calling approaches, and clustering thresholds. This helps prevent shifts in relatedness interpretation that could misinform epidemiologic inference (see Section 3). Recent reviews of long-read metagenomics have outlined practical pipeline elements and performance considerations to inform validation [97]. Recent ONT evaluations, including positive BC workflows and adaptive sampling, provide practical accuracy, depth, and turnaround benchmarks that can guide validation and quality-control expectations [85].

**Figure 1:**
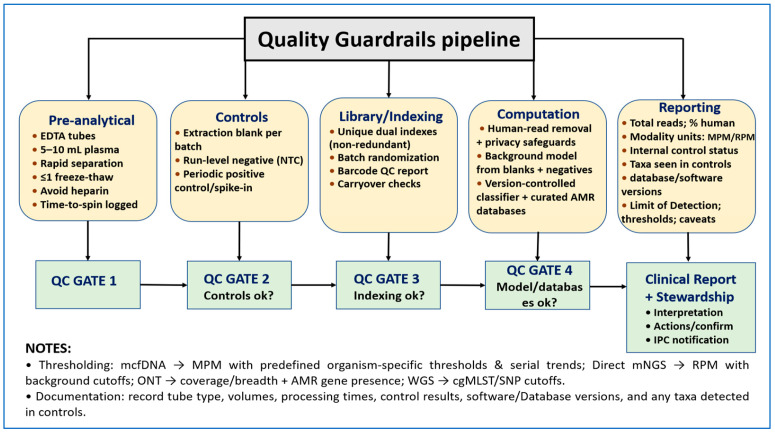
Quality controls and mitigation measures for blood/plasma sequencing, from preanalytics to reporting. Preanalytics (EDTA tubes; 5–10 mL plasma; rapid separation; more than or less 1 freeze–thaw; avoid heparin) flow into batch controls (extraction blanks, run-level negatives, periodic positive controls), library/indexing mitigation measures (UDI), batch randomization), and computation (human-read removal, contamination-aware background models, fixed, documented versioned classifiers with curated AMR databases, modality-specific quantitative thresholds), culminating in a standardized clinical report (total reads, percent human, modality units, MPM/RPM, control status, taxa in controls, software/database versions, limits of detection, cgMLST/SNP cutoffs) that supports stewardship decisions.

The regulatory environment continues to evolve. Although most infectious disease mNGS assays are currently offered as laboratory-developed tests under CLIA and CAP accreditation, regulatory agencies have issued draft guidance for targeted and metagenomic NGS devices that outlines recommended analytical validation studies. Harmonizing local validation plans with these recommendations and clearly linking quantitative thresholds to AMS actions in the report may simplify eventual in vitro diagnostic (IVD) transitions. This can be achieved without compromising near-term clinical utility. Figure 1 summarizes the quality pathway from preanalytical steps through batch controls, library preparation, indexing mitigation measures, contamination control, and standardized reporting. These elements support reliable blood and plasma sequencing and guide AMS decisions.

### 3.5. TAT and Where Each Method Fits

**Figure 2:**
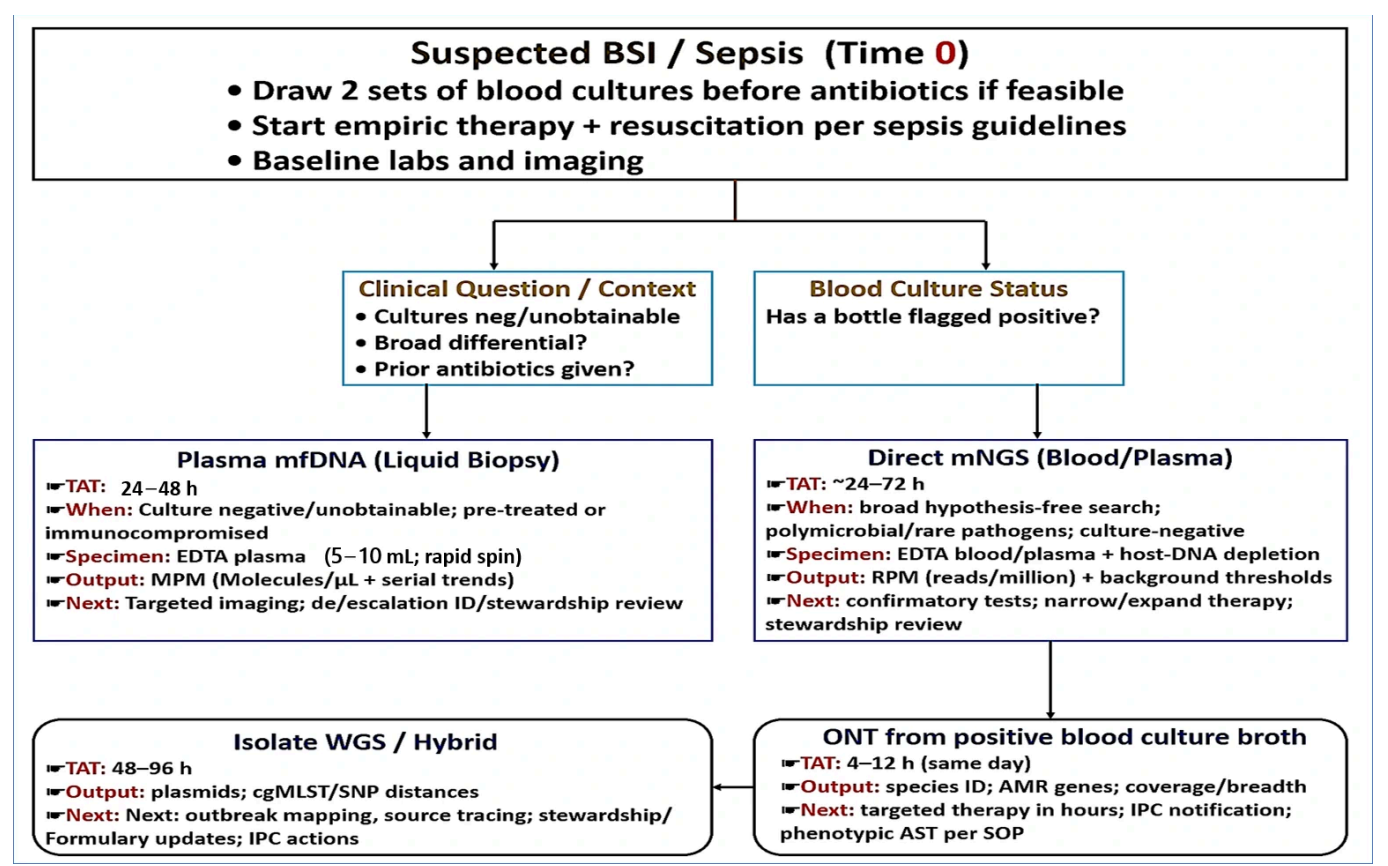
Practical decision pathway for sequencing in suspected BSIs. After drawing BCs and starting empiric therapy, select the sequencing modality according to the clinical context. Plasma mcfDNA supports broad, minimally invasive detection and burden trending when cultures are negative or unobtainable, or when early antibiotics are likely to depress culture yield (TAT 24–48 h). Direct mNGS of blood or plasma enables hypothesis-free discovery, including polymicrobial or unusual pathogens (TAT 24–72 h). When a BC flags positive, ONT sequencing from positive broth provides same-day species identification and on-target AMR gene calls (TAT 4–12 h). Isolate WGS or hybrid sequencing may then follow to define plasmid and resistance context and to support epidemiologic investigations (TAT 48–96 h).

This subsection summarizes typical TATs, pathogen scopes, and clinical situations in which each sequencing approach is most appropriate. Figure 2 and Table 2 provide comparative reference points and should be interpreted alongside this brief overview. Plasma mcfDNA testing typically delivers results within 24–48 h and reports quantitative values in MPM. It is most helpful when cultures are negative or unobtainable, after early antimicrobial exposure, or when serial MPM measurements assist in evaluating treatment response or localizing an infection source. Initial analytical and clinical validation demonstrated detection of approximately 1250 organisms with quantitative output, and a large real-world cohort of more than 15,000 patients showed broad organism recovery and clinically meaningful impact, supporting its use in culture-negative sepsis evaluation [21,22]. Many programs aim to provide actionable reports within 48 h [86].

Direct metagenomic sequencing of blood or plasma generally requires 24–72 h, depending on the extraction workflow, host-DNA depletion, sequencing depth, and computational pipeline. Published experience places the average TAT at approximately 48 h, although optimized protocols can deliver results in 6–24 h. This method is most appropriate when the differential diagnosis is broad, including polymicrobial or unexpected pathogens, or when prior antibiotics reduce culture sensitivity [18].

Once a BC signal is positive, ONT sequencing can compress species identification and resistance prediction into same-day reporting. Prospective ICU studies demonstrate species-level accuracy within hours directly from broth, along with detection of clinically relevant AMR loci and plasmids. Adaptive sampling can further enrich pathogen reads and improve sequencing depth [24]. To support rapid turnaround, many institutions implement predefined workflows coordinated with stewardship teams, enabling same-day de-escalation, escalation, or targeted source evaluation. In practice, laboratories often combine these approaches. Plasma mcfDNA or direct metagenomic sequencing supports culture-negative or pretreated cases, whereas ONT applied to positive BCs enhances organism identification and AMR profiling for early clinical action and outbreak recognition [24].

Figure 2 outlines a pragmatic time-to-result pathway integrating plasma mcfDNA, direct metagenomics, and ONT, followed when needed by isolate WGS. Approximate TATs, clinical indications, and stewardship actions are shown. Table 2 expands the comparison through specimen requirements, TATs, organism scopes, quantitative reporting, optimal clinical scenarios, strengths and limitations, and associated stewardship actions.

## 4. Applications of NGS in BSIs

While molecular assays (for example, shotgun metagenomic sequencing and mcfDNA testing) can accelerate pathogen detection and identify fastidious or uncultivable organisms, they do not replace conventional culture, which remains essential for phenotypic antimicrobial susceptibility testing, confirmatory identification, and epidemiologic and outbreak investigations. These modalities should therefore be regarded as complementary rather than interchangeable [19,98].

### 4.1. Bacterial BSIs: Rapid Pathogen ID and AMR Profiling

For common Gram-positive and Gram-negative BSIs, WGS and rapid sequencing from positive BCs provide same-day species identification, lineage assignment, and genotypic resistance calls that can inform treatment decisions. In methicillin-resistant *Staphylococcus aureus* (MRSA) bacteremia, WGS reliably resolves transmission routes and clonal lineages (e.g., MRSA ST22, ST239), revealing outbreaks that routine typing can miss. This approach can also shorten the time to infection-control action [99]. In *Enterococcus faecium*, ward-level surveillance uses WGS to confirm *vanA*/*vanB* carriage and delineate clusters such as ST78. These data also help benchmark transmission and guide contact-precaution measures [100].

In Enterobacterales, WGS clarifies the plasmid context of major carbapenemases (*bla*KPC, *bla*NDM, *bla*OXA-48-like) and tracks their movement across species and facilities. These insights inform cohorting strategies and infection control interventions at the health-system level. Multiyear surveillance highlights recurrent plasmid–strain combinations, particularly in *Klebsiella pneumoniae* (*K. pneumoniae*) CG258/ST258 and related clones [101]. These conclusions are supported by outbreak and network studies. Snitkin et al. reconstructed transmission during a hospital outbreak of KPC-producing *K. pneumoniae*. Spencer et al. demonstrated interfacility spread across an integrated healthcare network, and Han et al. identified patient-transfer-linked drivers of dissemination involving long-term care facilities [102,103,104]. WGS also separates clonal spread from plasmid-mediated dissemination. Marimuthu et al. quantified “hidden” transmission of carbapenemase-producing Enterobacterales via mobile elements, whereas Tsukada et al. documented a pediatric ward outbreak driven by cross-species plasmid movement [105,106].

In addition to carbapenem-resistant Enterobacterales (CRE), population-level genomics informs empiric therapy and stewardship. In England, a decade-long survey revealed a largely stable *Escherichia coli* (*E. coli*) BSI population punctuated by the rise of ST131; in Wales, ST131 bacteremias clustered geographically with predictable third-generation cephalosporin resistance; and a ten-year Oxfordshire series used WGS to refine outbreak recognition and link resistance to specific lineages [107]. For nonfermenters, WGS distinguishes importation from intrahospital transmission and links resistance to discrete genetic events. ICU investigations of *Pseudomonas aeruginosa* (*P. aeruginosa*) revealed prolonged, simultaneous outbreaks driven by high-risk clones ST111 and ST235 and tolerance to quaternary ammonium compounds, prompting focused environmental remediation [108]. In *Acinetobacter baumannii* (*A. baumannii*), long-horizon genomics showed decade-long persistence of OXA-24/40-producing clones within single hospitals [109].

Genotypic resistance predictions should be reconciled with local MICs and clinical responses because genotype and phenotype correlation vary by organism and drug pair. In practice, these data support earlier escalation or de-escalation (for example, carbapenem sparing when an ESBL is present without a carbapenemase, or earlier linezolid or daptomycin use when *vanA* or *vanB* is detected) and faster closure of suspected outbreaks using cgMLST and SNP thresholds. A pragmatic workflow pairs rapid ONT sequencing from positive bottles for same-day species identification and on-target resistance calls with short-read or hybrid assemblies when high-resolution plasmid structure and full resistome context are needed [24,37,108].

### 4.2. Viral BSIs

Sequencing underpins antiviral stewardship in major viral BSIs. In HIV, U.S. guidelines recommend genotypic resistance testing for virologic failure of reverse transcriptase, protease, and integrase inhibitors, and when an integrase strand transfer inhibitor (INSTI) regimen fails, as well as in defined baseline scenarios, because the detected mutations directly inform therapy. A 2024 systematic review and meta-analysis in antiretroviral-naive (ART-naive) individuals found that NGS detects pretreatment drug resistance more sensitively than Sanger sequencing, underscoring the clinical importance of low-abundance variants [110].

Earlier pooled analyses showed that minority non-nucleoside reverse transcriptase inhibitor (NNRTI)-resistant variants increase the risk of failure with NNRTI-based regimens. Population-based cohorts also linked NGS-defined pretreatment resistance to poorer outcomes, providing evidence for sensitive baseline genotyping in selected settings [111]. Deep sequencing has also mapped subtype diversity and resistance patterns after first-line failure in high-burden regions, demonstrating epidemiologic value beyond single-patient care [112].

For HBV, society guidance remains central; genotyping and resistance assessment inform drug choice and monitoring [113,114]. Compared with legacy assays, NGS can be validated for routine resistance testing and genotyping, with improved sensitivity for mixed populations [115]. A comprehensive review of putative tenofovir resistance showed that bona fide resistance is uncommon and often requires combinations of substitutions, reinforcing the value of high-depth sequencing to characterize minority resistance haplotypes when breakthrough is suspected [116].

For HCV, the American Association for the Study of Liver Diseases/Infectious Diseases Society of America (AASLD/IDSA) guidance recommends baseline nonstructural protein 5A (NS5A) resistance-associated substitution (RAS) testing in genotype 3 for specific regimens. Mutations such as Y93H can alter first-line choices, including ribavirin addition or the use of alternative direct-acting antivirals [117,118]. NGS provides sensitive NS5A detection at low frequency, which can be decisive in previously treated or cirrhotic patients.

For cytomegalovirus (CMV) in immunocompromised hosts, targeted sequencing of UL97 and UL54 (and newer panels including UL56 and UL27) is increasingly used when plasma DNAemia persists despite therapy. Sahoo et al. developed and analytically validated an amplicon-based assay in clinical plasma that detects low-abundance resistance mutations with a defined error model, enabling early recognition of ganciclovir or foscarnet failure [119]. In a nationwide solid-organ transplant cohort, López-Aladid et al. reported that NGS outperforms Sanger sequencing for detecting resistance and that the results correlate with outcomes, demonstrating real-world clinical impact [120]. Recent panels covering multiple resistance genes have been validated for clinical use, enabling broader and faster workups in refractory viremia [121]. In addition, long-range PCR coupled with sequencing has been introduced to read all resistance-associated genes concurrently and detect low-frequency variants earlier [122].

For SARS-CoV-2, RNAemia is a robust prognostic biomarker. A large U.S. multicenter study showed that plasma viremia correlates with greater disease severity and mortality and outperforms many inflammatory markers [123]. Other cohorts have shown that RNAemia is common and associated with worse outcomes, aiding risk stratification [124]. ICU analyses have further linked RNAemia to higher 28-day mortality, demonstrating how quantitative bloodstream viromics can augment clinical prediction [125]. Additional hospital cohorts similarly link serum RNA detection with mortality and immune dysregulation [126].

For major viral pathogens such as HIV, HBV, HCV, CMV, and SARS-CoV-2, sequencing of viral nucleic acids in blood or plasma provides highly actionable information. This includes resistance genotypes that prompt changes in antiviral therapy and RNAemia measures that support prognosis, triage, and monitoring. The shared operational lesson is that analytical depth and validated cutoffs matter. Low-frequency variants and quantitative viremia carry clinical weight only when they are generated and interpreted within protocolized, quality-controlled workflows that are linked to clear downstream actions [113,120].

### 4.3. Fungal BSIs

For yeasts and molds, targeted sequencing and WGS complement culture by resolving species, detecting resistance mechanisms, and clarifying transmission. In candidaemia, species-level identification directs therapy (for example, *Nakaseomyces glabrata* versus *Candida parapsilosis*), and sequencing underpins surveillance of *Candida auris*, an emerging healthcare-associated yeast that frequently shows azole resistance via ERG11 substitutions and echinocandin resistance via FKS1 hotspot mutations. Early global genomics revealed nearly simultaneous emergence of *Candida auris* on three continents with distinct clades by WGS, establishing the need for genomic epidemiology alongside routine antimicrobial susceptibility testing [127,128]. Subsequent work described clade-specific structural variation and local outbreak genomics linking patient-to-patient transmission and facility introductions, justifying unit-level interventions and environmental controls [129,130].

In non-auris *Candida* species, sequencing resolves resistance patterns that directly influence clinical management. In *Candida parapsilosis* candidaemia, ERG11 Y132F has emerged as a dominant mechanism of fluconazole resistance across multiple regions and has been repeatedly associated with clonal outbreaks, supporting early azole-sparing regimens and focused infection-prevention measures [131,132]. In *Nakaseomyces glabratus*, candidaemia, echinocandin resistance correlates with FKS1 or FKS2 hotspot mutations. Clinical cohorts link these variants to elevated MICs and worse outcomes, supporting rapid switches to alternative therapy when resistance genotypes are detected [133,134].

In molds, azole resistance in *Aspergillus fumigatus* is commonly driven by cyp51A promoter and target alterations, particularly TR34/L98H and TR46/Y121F/T289A, which can arise through environmental fungicide exposure and have been documented in clinical disease and environmental reservoirs worldwide. Sequencing detects these alleles in patient and environmental isolates and links them to azole treatment failure, guiding the selection of amphotericin B or combination therapy and informing One Health policy on agricultural azoles [135,136,137].

For *Cryptococcus* species, WGS separates the *Cryptococcus neoformans* and *Cryptococcus gattii* complexes and refines within-species lineages that correlate with geography, phenotype, and outcome. Population genomic studies within Africa, Asia, and the Americas map dominant lineages and transmission patterns [138,139]. When 5-flucytosine resistance emerges during therapy, sequencing can reveal mutations in the pyrimidine salvage pathway, such as FUR1, FCY1, and FCY2, or other mechanisms, supporting early modification of 5-flucytosine-containing regimens [140,141,142].

### 4.4. Polymicrobial and Culture-Negative Infections

Unbiased metagenomic sequencing, whether performed directly on blood or plasma or by measuring mcfDNA, is most useful when cultures are negative. It is also valuable when prior antibiotics are likely to suppress viability or when the differential diagnosis spans bacteria, viruses, and fungi, including mixed infections. Large real-world and programmatic series demonstrate broader pathogen detection and actionable results in a meaningful subset of patients. These findings are most reliable when interpreted within contamination-aware workflows and multidisciplinary reviews [22,143,144]. Prospective and early-use studies likewise report accelerated diagnosis and antimicrobial changes compared with standard testing [145].

Head-to-head comparisons indicate that plasma metagenomic sequencing can have higher or complementary sensitivity relative to BC in suspected BSI, improving detection of fastidious or partially treated pathogens [146,147]. Matrix choice matters: a comparative analysis showed that plasma mcfDNA outperforms cellular fractions for detection and is less affected by host DNA, informing specimen selection in clinical workflows [148]. In bacteremia and infective endocarditis, plasma mcfDNA signals often persist longer than culture positivity after antibiotic exposure, aiding adjudication of partially treated disease and supporting planning of source-control evaluations [149].

Building on the time-to-result framework described in Section 3.5 and Figure 2, these modalities are best viewed as coordinated components of a single diagnostic pathway rather than standalone tests. In polymicrobial or culture-negative presentations, plasma mcfDNA and short-read metagenomics extend detection beyond conventional BC, whereas rapid sequencing from positive BCs supports early resistance profiling and epidemiologic investigation. Programs that embed these integrated workflows within diagnostic stewardship and infection-prevention pathways report the clearest clinical gains [24,37]. Table 3 translates these integrated workflows into disease-specific use cases, pairing exemplar pathogen groups with the preferred specimen and sequencing approach, actionable outputs (such as species identification, resistance markers, and epidemiologic metrics), and expected TATs.

## 5. Technical and Translational Challenges

### 5.1. Sample Preparation (Low Microbial Load, Host-DNA Depletion, and Preanalytics)

Blood is a low-biomass matrix dominated by human DNA, and without careful preanalytical control and enrichment, microbial signals can be missed. Foundational clinical metagenomics studies emphasize specimen integrity, host read subtraction, and orthogonal confirmation to ensure analytical validity and clinical utility [18]. Practical wet-lab strategies include differential lysis with nuclease treatment for host DNA depletion and optimized library preparation. Experience from another low-biomass setting (the lower respiratory tract) shows that nanopore-based workflows incorporating depletion can deliver actionable results within hours, underscoring that upstream processing strongly influences sensitivity and TAT [151].

Preanalytical factors also influence how much mcfDNA is recovered by mNGS. Compared with cellular samples or tissues, cell-free fluids such as plasma contain less host nucleic acid, so a greater share of reads comes from microbes, and sensitivity improves. To maintain this advantage, samples should be handled carefully, with steps to limit background and contamination and to preserve stability during storage and transport. At the same time, cfDNA approaches may be less sensitive for mainly intracellular pathogens and do not capture cell-based host-response signals, so these limitations should be kept in mind when interpreting results [18].

EDTA is preferred because heparin inhibits polymerase chain reaction (PCR), a classic finding that still applies to culture-independent diagnostics [57]. Stakeholder frameworks have formalized expectations. The Blood Profiling Atlas in Cancer Consortium (BloodPAC) validation protocols and the Minimum Preanalytical Data Elements (MTDEs) define required fields and process checks, with recent updates for liquid biopsy applications [152,153]. Complementary guidance from the National Cancer Institute Biospecimen Evidence-Based Practices outlines standard operating procedures for specimen collection, processing, and storage. Many laboratories adopt or adapt these procedures when validating plasma-based infectious-disease assays [154]. These preanalytical and low-biomass considerations support the direct metagenomic workflows described in Section 3.2.2. They should be applied consistently across blood- and plasma-based assays to ensure reliable quantitative interpretation.

### 5.2. Bioinformatics Variability (Classifier Choice, AMR Annotation, and Contamination Control)

Downstream data analysis is as important as specimen processing. Widely used metagenomic classifiers differ in speed, memory use, and precision; for example, Kraken2 (exact k-mer matching) is very fast, whereas Centrifuge (FM-index-based) is more memory-efficient. Therefore, laboratories should fix software versions, reference builds, and decision thresholds during validation and reevaluate them after any meaningful updates [80,91]. For AMR annotation, resources differ in their curation strategies. AMRFinderPlus (NCBI) maps genes and point mutations with strong phenotype concordance for many organism and drug pairs, whereas CARD with RGI and ResFinder/PointFinder offer complementary coverage. Reports should disclose the database used, the identity and coverage thresholds, and any species-specific mutation rules to support reproducibility and peer review [83,155].

In practice, much of the interlaboratory variability in bloodstream metagenomics arises not from sequencing chemistry but from different choices of classifiers, databases, background models, and reporting thresholds. As detailed in Section 3.4, contamination-aware workflows and low-biomass specific controls are essential for accurate interpretation. However, laboratories often use different limits of detection, index-swap mitigation strategies, and pass/fail criteria, which can make results difficult to compare. Aligning validation plans with published frameworks such as RIDE, other low-biomass guidance, early clinical metagenomics consensus documents, and STROBE metagenomics reporting standards, and explicitly documenting contamination screen criteria and quantitative cutoffs can narrow this gap. These steps also help facilitate meaningful cross-center comparisons [33,65]. In addition, regulatory-grade reference resources such as the Food and Drug Administration Antibiotic Resistance Genomic Sequence Database (FDA-ARGOS) provide vetted genomes that support assay development and reduce the risk of misclassification associated with imperfect databases. These resources complement local validation efforts [85].

### 5.3. Interpretation Hurdles (Infection vs. Colonization vs. Background)

Even with robust pipelines, interpreting positive bloodstream results requires a clinical context. Expert reviews emphasize integrating quantitative readouts with antibiotic timing, host status, imaging, and conventional microbiology to distinguish true infection from transient translocation or background, especially when values approach the laboratory’s background model [18]. Real-world cohorts using plasma mcfDNA quantification (MPM) show how burden trends can help assess treatment response and prioritize diagnostic evaluation. These studies also remind clinicians to balance broad detection with stewardship principles [22]. Incorporating validated reference genomes such as FDA-ARGOS and using fixed, documented classifiers improves specificity by reducing taxonomic misassignment at low read counts [85].

### 5.4. Economic and Regulatory Barriers (Cost, TAT, Reimbursement)

TAT and cost shape how sequencing is integrated into routine care. In the United States, most infectious disease sequencing assays are offered as CLIA/CAP laboratory-developed tests that follow metagenomic validation guidance and STROBE-metagenomics reporting standards while navigating evolving payer policies [33]. The American Medical Association (AMA) Proprietary Laboratory Analyses (PLA) coding system now includes codes for specific infectious disease sequencing tests, such as 0152U for a plasma mcfDNA assay, creating a structured billing pathway but not guaranteeing payment [156]. CMS gap-fill deliberations summarize the scope of the test (more than 1000 detectable organisms) and inform pricing decisions, illustrating how reimbursement is determined for novel diagnostics [21].

Commercial coverage varies and often depends on clear evidence of clinical utility and well-defined indications. Several major policies specifically address plasma mcfDNA or metagenomic sequencing for culture-negative evaluations [157]. As reimbursement frameworks mature, aligning validation with FDA reference initiatives such as FDA-ARGOS and with recognized preanalytical and analytical frameworks, including BloodPAC and Clinical Chemistry guidance, may help prepare assays for future in vitro diagnostic pathways. This alignment can also support more predictable coverage decisions [85,152].

Sequencing coexists with rapid, lower-cost PCR systems that are already available. FDA-cleared multiplex BC panels such as BioFire BCID2 and ePlex identify common BSIs and key resistance determinants (including *mecA/C*, *vanA*/*vanB*, and carbapenemases such as *bla*KPC, *bla*NDM, and *bla*OXA-48-like) directly from positive bottles, with reported times to result of about 1 h. This rapid turnaround aligns well with emergency decision-making and AMS pathways [158,159]. These panels have longstanding FDA 510(k) clearance for Gram-positive, Gram-negative, and fungal targets and are widely deployed. Their established use enables predictable reimbursement and easier protocol integration compared with newer sequencing workflows [160]. In contrast, NGS modalities usually require more time and resources. Plasma mcfDNA results are typically reported within 24 to 48 h in programmatic series. Even rapid nanopore sequencing from positive broth usually yields actionable calls within the same shift rather than within the first hour, reflecting the additional wet-lab and bioinformatics steps involved [160,161].

Moreover, per-test costs and infrastructure requirements (sequencers, validated pipelines, trained personnel) remain higher for metagenomics than for PCR panels. Implementation reviews and cost analyses highlight this gap, which slows adoption outside specialized centers [15,162,163]. These realities explain current practice. PCR panels are typically the first-line rapid tool once a bottle flags positive, whereas NGS adds value as an adjunct for culture-negative workups, broader organism scope, resistance epidemiology, and outbreak resolution. Continued improvements in sequencing and analysis are expected to further compress TAT and reduce the total cost of ownership [164].

## 6. Future Perspectives

Advances in machine learning assisted interpretation and portable sequencing are making mNGS faster to run, simpler to operationalize, and easier to interpret in clinical settings. For taxonomic classification, deep learning models are emerging as adjuncts to reference-based tools. DeepMicrobes learns species-level signals directly from raw reads and improves precision and recall on shotgun datasets, while architectures such as MetageNN are designed to tolerate long-read error profiles and incomplete reference catalogs, which is useful when data are sparse or noisy [165,166].

At the bedside, sepsis risk prediction using artificial intelligence is promising, but it requires rigorous external validation, transparent performance reporting, and careful clinical oversight before it is used to guide decisions. A rigorous external evaluation of the widely deployed Epic Sepsis Model showed poor discrimination and substantial missed cases in routine care. These findings underscore the need for prospective, local validation before the model is used to guide clinical decisions. In parallel, reinforcement learning policies and early warning systems have demonstrated feasibility for individualized triage in research settings. The practical lesson is that strong governance, fixed and documented software versions, monitoring for model drift, and explicit action thresholds are essential. These practices are preferable to implementing models without careful evaluation [167].

Real-time nanopore sequencing has shown field utility during Ebola and Zika responses and is increasingly being integrated into hospital workflows. ICU and emergency settings can now deliver same-day organism identification and targeted AMR calls directly from positive BC broth. Multicenter efforts continue to define accuracy and validation requirements for cgMLST and SNP typing. Ongoing improvements in reagent chemistry and adaptive sampling are making true bedside sequencing increasingly plausible, particularly as a rapid pathway once a bottle flags positive [168].

In parallel, plasma mcfDNA testing has progressed from initial validation to scaled clinical use. An index study established quantitative detection for nearly 1200 pathogens. Large programmatic series involving more than 15,000 patients have shown added yield in culture-negative or pretreated sepsis, with signal kinetics that can persist after antibiotic treatment. Consistent with the premise in Section 4 that sequencing complements rather than replaces BC, the near-term role of plasma mcfDNA is as an adjunct and triage tool when cultures are negative or unobtainable or for burden trending. Any move toward partial replacement of BC would require outcome-focused trials demonstrating noninferiority for source control and AMS endpoints [165,169].

Genome-enabled surveillance is expanding from single centers to coordinated, cross-sector networks. The WHO Global Antimicrobial Resistance and Use Surveillance System (GLASS) is broadening standardized surveillance and explicitly encourages the use of genomic methods. Community platforms such as Pathogenwatch enable lineage-resolved tracking, including for *Klebsiella* and other priority pathogens.

These tools integrate built-in public health analytics to support surveillance and infection-control decision-making. The WHO Global Genomic Surveillance Strategy establishes governance structures and data-sharing norms for genomic surveillance. Over time, these frameworks will increasingly incorporate bloodstream pathogens from human, animal, and environmental reservoirs. The operational implications for hospitals run in both directions. Local WGS data feed global situational awareness, while the global context, including clade- and lineage-specific resistance patterns, guides empiric therapy and outbreak preparedness at the bedside [170,171].

## 7. Conclusions

NGS has moved from research into routine care for BSIs and sepsis. It enables rapid, culture-independent pathogen detection, broad AMR profiling, and genome-resolved epidemiology that shorten the time to effective therapy, support stewardship, and strengthen infection-prevention programs. Complementary modalities now address distinct clinical scenarios: plasma mcfDNA for minimally invasive quantitative assessment, direct mNGS of blood or plasma for hypothesis-free detection in culture-negative or polymicrobial disease, and same-day nanopore sequencing of positive BC broth to accelerate species identification and targeted resistance calls.

Nevertheless, important challenges remain. Low microbial biomass, high background human DNA, and exogenous contamination can blunt sensitivity, and variability in bioinformatics choices and reference curation can impair reproducibility. Furthermore, distinguishing infection from colonization or background requires careful synthesis with laboratory data, imaging, and bedside findings. Regulation, cost, and reimbursement also influence adoption. However, the near-term progress is notable. Model-assisted analytics, point-of-care and portable sequencing, and scaled liquid-biopsy strategies are poised to further decrease TATs and expand access. Embedding sequencing within One Health and global genomic surveillance networks should improve early detection of emerging resistance and enable coordinated response.

Realizing the full clinical impact will require ongoing technical innovation. Rigorous preanalytical practice with stringent contamination control and effective host-DNA depletion will also be necessary, as will stable version-controlled software and database builds with validated pipelines and quantitative thresholds. Implementation studies linked to patient-centered outcomes should also be conducted to advance sepsis care from empiricism toward precision medicine.

## Figures and Tables

**Table 1:** Evidence-supported Bioinformatics Toolkit for Bloodstream Sequencing.

Task	Example Tools/Workflows	Primary Outputs	Key Notes	Key References
Human-read subtraction/alignment	Burrows Wheeler Aligner (BWA-MEM); Bowtie 2; minimap2	Alignment of reads to large references; host-filtered BAM/FASTQ	BWA-MEM aligns short–long reads; Bowtie 2 fast gapped alignment; minimap2 supports long/short reads	[87,88,89]
Taxonomic classification	Kraken 2 with Bracken; Centrifuge	Taxonomic labels; abundance estimates	Kraken 2 high accuracy and low memory; Bracken re-estimates abundance; Centrifuge rapid classification	[80,90,91]
Background/contamination modeling	Negative control filtering; decontam (R)	Contaminant identification	Low biomass reagent contamination; decontam uses statistical models	[59,92]
AMR gene/variant detection	AMRFinderPlus; CARD/RGI; ResFinder; PointFinder	AMR gene and mutation detection	AMRFinderPlus detects genes & mutations; CARD curated families; ResFinder phenotype prediction; PointFinder mutation detection	[82,83,84,93]
Quality control summaries	FastQC; MultiQC	QC metrics; aggregated QC	FastQC QC checks; MultiQC aggregates	[94]
Index-swap mitigation	UDI	Reduced index hopping	UDI enables removal of swapped reads	[65,73]
Genome typing/outbreak relatedness	ONT positive BC; Guppy; Medaka; Flye	Rapid ID; assemblies; AMR detection	Rapid ID & AMR detection from BC	[24,37]
Viral variant calling	iVar; LoFreq	Consensus + variant calls	iVar amplicon framework; LoFreq low-frequency variant calling	[95,96]
Clinical reporting	Version-annotated reporting; STROBE-metagenomics	Structured reports	Improves transparency and reproducibility	[85]

**Table 2:** Sequencing modalities for suspected BSI: specimen, TAT, use cases, and operational notes.

Modality	Specimen and Minimum Volume	Typical TAT	Organism Scope	Quantitative Output	Best-Fit Clinical Scenarios	Key Strengths	Main Limitations and Pitfalls	Typical Stewardship Actions
Plasma mcfDNA	EDTA plasma, 5–10 mL; rapid separation; ≤1 freeze–thaw (e)	24–48 h (a)	Bacteria (DNA), DNA viruses, fungi, parasites; around 1, 250 targets (a)	MPM (molecules/µL)	Culture negative or unobtainable; pretreated; immunocompromised; fastidious or occult pathogens; quantitative burden trending	One-test breadth; quantitative trending; minimally invasive	Nonlocalizing; no phenotypic MICs; residual or nonviable DNA; low-biomass contamination risk (f)	Protocolized escalation or deescalation based on pathogen and MPM trend; targeted imaging and source evaluation; ID stewardship
Direct mNGS plasma	EDTA plasma, 3–10 mL; hostDNA depletion recommended (e)	24–72 h (b,c)	Broad, hypothesis-free	RPM or unique k-mers; relative abundance	Wide differential, including polymicrobial or unusual pathogens; culture negative or delayed	Unbiased detection; complements culture and panels	Low microbial biomass; contamination/background modeling required (f); thresholds needed	Narrow or expand therapy for high-confidence hits; order confirmatory tests; ID consult
Direct mNGS whole blood/cellular fraction	Whole blood 1–5 mL; optimized extraction for cellular fraction (e)	24–72 h (c)	Broad; may enrich intracellular/pathogen DNA in cells	RPM or unique k-mers	When cellular fraction may add yield; complementary to plasma testing	Complementary to plasma; may capture different taxa	Higher host background; matrix-dependent performance; contamination and index controls (f)	As above; reconcile with plasma results and clinical context
Positive BC ONT	Positive BC broth (direct DNA)	4–12 h (same-day) (d)	Primarily bacteria (from BC)	Depth/coverage; AMR gene presence	BC flagged positive; rapid ID/AMR; plasmid/resistance-context; rapid epidemiology	Same-day ID and genotypic AMR; plasmid context; supports rapid epidemiology	Requires culture positivity; genotype- phenotype for some pairs; thresholds must be validated	Targeted therapy within hours; infection-prevention notification; phenotypic confirmation per protocol
Targeted sequencing 16S rRNA	DNA from specimen or BC isolate	24–48 h (g)	Bacteria (barcode)	Qualitative (ID call)	Culture negative; slow growing/fastidious; polymicrobial clarification	Broad bacterial ID; low input	Limited species-level resolution in some genera; copy number bias; database dependence	De-escalate; confirm unusual taxa; plan targeted cultures
Targeted sequencing ITS (fungi)	DNA from specimen or BC isolate	24–48 h (g)	Fungi (barcode)	Qualitative (ID call)	Candidemia and other invasive mycoses; mixed fungal infections	Species-level calls that guide antifungal selection	Primer bias; molds/cryptic yeasts may need D1/D2, TEF1, β-tubulin (g)	Optimize antifungal choice and duration; epidemiologic linkage
Targeted AMR panels (bacterial and fungal)	DNA from specimen or positive BC	6–24 h (g)	Focused AMR loci (*bla*ESBL, carbapenemases; *vanA*/*vanB*; ERG11; FKS1/FKS2)	Gene/allele calls	Specific mechanisms suspected; need rapid resistance information	Fast and actionable; high depth over key loci	Panel limited; may miss off-panel mechanisms; genotype-phenotype gaps	Rapid escalation or de-escalation; isolation precautions for high-risk genes
Hybrid short + long read assemblies (outbreaks/plasmids)	DNA from isolate or positive BC	1–3 days (h)	Bacterial genomes; plasmids	Closed/near closed assemblies	Outbreak resolution; plasmid and AMR-context mapping	Most reliable plasmid reconstruction; mobile-element context	More resources and time; specialized bioinformatics	Infection-prevention interventions; source tracing; stewardship and formulary updates

Footnotes: (a) Validation of quantitative MPM and organism breadth for plasma mcfDNA: [21]. (b) Large real-world mcfDNA series and clinical impact (more than 15,000 tests): [22]. (c) Typical direct mNGS turnaround in clinical workflows: [18]. (d) Same-day ONT species/AMR from positive BCs; adaptive sampling: [24,37]. (e) Preanalytics for blood/plasma (tube choice, volume, processing time, freeze–thaw): [55,56,57]. (f) Low biomass contamination controls and RIDE checklist; index hopping mitigation: [33,65]. (g) Targeted assays of 16S, ITS, and AMR panels in clinical practice [29,44]. (h) Hybrid assemblies for plasmid/AMR context and outbreak mapping: [29,44].

**Table 3:** NGS in BSIs: Specimen, Method, Outputs, and TAT.

Pathogen Group (Example)	Clinical Question	Preferred Specimen	Sequencing Approach	Actionable Outputs	Typical TAT	Key References
MRSA	Outbreak investigation, lineage assignment, rapid identification and antimicrobial resistance	Positive BC broth	ONT from positive BC; WGS for final resolution	Species; MRSA lineage (for example ST22 or ST239); outbreak linkage	Hours for ONT; 24–72 h for WGS	[24,99]
VRE	*vanA* or *vanB* carriage and clustering	Positive BC	WGS	*vanA* or *vanB*; cgMLST clusters; transmission benchmarking	24–72 h	[100]
*K. pneumoniae* (CG258/ST258)	Carbapenemase context and spread	Positive BC	WGS or hybrid (short plus long reads)	*bla*KPC, *bla*NDM, *bla*OXA-48-like; plasmid context; network spread	48–72 h	[101,102]
*P. aeruginosa*	Importation versus transmission; resistance drivers	Positive BC	WGS	High risk clones; disinfectant tolerance; Verona integron encoded metallo-β-lactamase (VIM) and related determinants	48–72 h	[108]
*A. baumannii*	Persistence and clonality	Positive BC	WGS	OXA carbapenemases; ward-persistence mapping	48–72 h	[109]
HIV	Drug resistance at failure or baseline in selected settings	Plasma	Targeted NGS of pol (reverse transcriptase, protease, integrase)	Drug-resistance mutation profile informing regimen change	2–5 days	[110,111,150]
HBV	Genotype and resistance assessment	Plasma	Deep sequencing of polymerase	RAS; genotype guiding therapy	2–5 days	[113,115,116]
HCV (genotype 3)	Baseline NS5A RAS (for example Y93H)	Plasma	Targeted NGS of NS5A	RAS informing direct-acting antiviral selection	2–5 days	[117,118]
CMV	Resistance during DNAemia	Plasma	Amplicon panel covering UL97, UL54, UL56, UL27	Early detection of resistance informing switch of therapy	2–4 days	[119,120,121]
SARS-CoV-2	Prognosis based on RNAemia	Plasma	Quantitative PCR or sequencing based quantification	Risk stratification and monitoring	Same-day to 48 h	[86,123,125]
*Candida auris*	Clade assignment and azole or echinocandin resistance	Positive BC	WGS with or without targeted ERG11 and FKS1	Clade identification; resistance markers guiding therapy and infection-prevention control	48–96 h	[127,128,130]
*Candida parapsilosis*	Fluconazole resistance mechanism	Positive BC	Targeted ERG11 amplicon sequencing	Y132F detection guiding azole-sparing therapy	24–72 h	[131,132]
*Nakaseomyces glabratus*	Echinocandin resistance mechanism	Positive BC	Targeted FKS1 and FKS2 sequencing	Hotspot mutations guiding change in therapy	24–72 h	[133,134]
*Aspergillus fumigatus*	Azole resistance mechanism	Blood rarely; culture or bronchoalveolar lavage when available	Targeted cyp51A or WGS	TR34/L98H and TR46/Y121F/T289A informing therapy choice	48–96 h	[135,136]
*Cryptococcus* species	Lineage assignment and 5-flucytosine resistance	Positive BC or CSF	WGS; targeted FUR1, FCY1, FCY2	Lineage assignment; resistance mechanisms related to 5-flucytosine	48–96 h	[139,140,142]

## Data Availability

No new data were created or analyzed in this study. Data sharing is not applicable to this article.
